# A Diverse Repertoire of Exopolysaccharide Biosynthesis Gene Clusters in *Lactobacillus* Revealed by Comparative Analysis in 106 Sequenced Genomes

**DOI:** 10.3390/microorganisms7100444

**Published:** 2019-10-11

**Authors:** Dipti Deo, Dimple Davray, Ram Kulkarni

**Affiliations:** Symbiosis School of Biological Sciences, Symbiosis International (Deemed University), Lavale, Pune 412 115, India; dipti.deo@ssbs.edu.in (D.D.); dimple.davray@ssbs.edu.in (D.D.)

**Keywords:** heteropolysaccharides, Wzy-dependent pathway, PATRIC, lactic acid bacteria, ecological niche, hierarchical clustering

## Abstract

Production of exopolysaccharides (EPS) is one of the unique features of *Lactobacillus* genus. EPS not only have many physiological roles such as in stress tolerance, quorum sensing and biofilm formation, but also have numerous applications in the food and pharmaceutical industries. In this study, we identified and compared EPS biosynthesis gene clusters in 106 sequenced *Lactobacillus* genomes representing 27 species. Of the 146 identified clusters, only 41 showed the typical generic organization of genes as reported earlier. Hierarchical clustering showed highly varied nature of the clusters in terms of the gene composition; nonetheless, habitat-wise grouping was observed for the gene clusters from host-adapted and nomadic strains. Of the core genes required for EPS biosynthesis, *epsA*, *B*, *C*, *D* and *E* showed higher conservation, whereas *gt*, *wzx* and *wzy* showed high variability in terms of the number and composition of the protein families. Analysis of the distribution pattern of the protein families indicated a higher proportion of mutually exclusive families in clusters from host-adapted and nomadic strains, whereas those from the free-living group had very few unique families. Taken together, this analysis highlights high variability in the EPS gene clusters amongst *Lactobacillus* with some of their properties correlated to the habitats.

## 1. Introduction

*Lactobacillus* represents one of the most astonishing genera of bacteria. Members of this genus are associated with many fermented food products, are considered to be probiotic offering numerous health benefits to the host, have industrial applications for the production of chemicals such as lactic acid and have GRAS (generally recognized as safe) status [1,2]. Many of these useful properties and applications of lactobacilli are because of their peculiar features including production of exopolysaccharides (EPS), lactic acid, short chain fatty acids and antibacterial peptides, and ability to tolerate low pH and bile and to attach to the mammalian intestinal epithelial cells [3]. Thus, understanding the biochemical and genetic basis of these processes is important for further exploitation of these bacteria.

Numerous studies have reported the production of EPS by lactobacilli [4,5,6,7,8,9]. While the exact physiological function of EPS is not clearly understood, they appear to be involved in resistance towards various environmental stresses such as desiccation, bacteriophages, toxic compounds like metal ions, antibiotics, hydrolyzing enzymes, bile salt, high salt concentrations and varying pH [10,11,12,13]. In addition, EPS are involved in the attachment of lactobacilli to the intestinal cells in the host, disallowing the attachment of competing pathogenic bacteria to the host cells and promoting the growth of beneficial bacteria [14,15]. EPS are also an important component of biofilms produced by lactobacilli, which assist in keeping the bacterial cells in close proximity with each other further helping in horizontal gene transfer and developing synergistic microconsortia [10,16]. Considering all these functions of the EPS, mostly associated with the environmental interactions of lactobacilli, the properties of enzymes involved in their biosynthesis and their regulatory mechanisms are likely to be correlated to the environment where the producer lactobacilli are found.

EPS produced by lactobacilli also have technically useful properties and biological activities. In dairy products, EPS have a direct influence on the texture and rheological properties [17,18]. Many of the EPS also display immunomodulatory, antimicrobial, antioxidant, antibiofilm, antitumor, flocculating and emulsifying activities [19,20,21,22]. EPS also find food and cosmetic-related as well as clinical applications [23,24].

The EPS produced by *Lactobacillus* strains include both homo- and heteropolysaccharides. Biosynthesis of the former requires fewer enzymes, whereas that of the latter occurring via the Wzy-dependent pathway is conferred by gene clusters encoding the enzymes and other proteins involved in the assembly and transport of these polymers as well as the regulation of this process [25]. Gene organization and functional properties of the EPS gene clusters have been studied in some lactobacilli, such as *L. delbreuckii* [4], *L. helveticus* [26], *L. johnsonii* [8,10,27], *L. paraplantarum* [28], *L. plantarum* [29,30,31] and *L. rhamnosus* [6,32]. These studies have highlighted some common features of EPS gene clusters in *Lactobacillus* such as the presence of the regulatory genes on the 5’end, polymerization and export-related genes on the 3’end and glycosyltransferase (gt) genes at the center of the cluster (reviewed by Zeidan et al., 2017) [24]. Many differences in the EPS gene clusters of various species are also reported. Some species such as *L. johnsonii* and *L. helveticus* harbor a single cluster [8,10,26,27], whereas few species such as *L. plantarum* and *L. paraplantarum* have multiple clusters [28,29,30,31]. At the 5’end, the first five genes of the clusters including a transcriptional regulator, *epsA*; a phosphoregulatory module, *epsBCD*; and a priming glycosyltransferase, *epsE* are usually conserved and present in a specific order often referred to as *epsABCDE* stretch in generic EPS clusters [8,10,24,26,27]. In a few other clusters, only a few of these five genes are conserved at the same location, whereas in some clusters, the organization of genes in the clusters is mosaic [6]. Furthermore, EPS clusters were also found to be one of the most variable regions across the genomes of 54 strains of *L. plantarum* [33]. Collectively, these facts indicate a great extent of variation in the gene composition and organization of *Lactobacillus* EPS clusters.

Complete genome sequences for numerous *Lactobacillus* strains have recently become available. Considering this, and that the above-mentioned studies highlight some commonalities and discrepancies in the EPS gene clusters across various lactobacilli, as well as the importance of EPS, a comprehensive understanding of the diversity in the EPS clusters in *Lactobacillus* becomes important and feasible. In this study, we have analyzed the composition and diversity of EPS gene clusters in 106 strains of *Lactobacillus* for which complete genome sequences were available in the public databases.

## 2. Materials and Methods

Genome sequence and the information on the open reading frames in these genomes were obtained from the NCBI and PATRIC databases. Initially, protein sequences encoded by highly conserved genes reported in the characterized EPS clusters in *Lactobacillus* were used as a query for tblastn against the genome of the selected *Lactobacillus* strains for which the complete genome was available on the NCBI as well as PATRIC databases. The query sequences used include transcriptional regulator (EpsA), tyrosine kinase modulator (EpsB), tyrosine kinase (EpsC), phosphotyrosine phosphatase (EpsD) and priming glycosyltransferase (EpsE) and from *L. delbrueckii* subsp *bulgaricus* Lfi5, *L. rhamnosus* GG, *L. johnsonii* FI9785 and *L. plantarum* WCFS1. The genomic environments of the obtained hits were manually evaluated for the presence of EPS biosynthesis-related genes. This was achieved by evaluating the annotation of the adjoining genes in both the databases as well as subjecting them to BLAST (identity > 30%, E-value < 1e-15). This resulted in identification of the whole gene clusters.

The nucleotide sequences of the identified gene clusters were downloaded from the NCBI database in Genbank format and used for building the gene cluster using EasyFig program [34]. Information on the families (PLFam and PGFam) of the proteins predicted from the gene sequences was obtained from the PATRIC database, whereas that on the habitats of various *Lactobacillus* species was from Duar et al., 2017 [35]. Glycosyltransferase (GT) proteins were classified with the help of dbCAN2 server [36]. Of the three search tools implemented in this strategy, HMMER search against dbCAN HMM database, DIAMOND search against the CAZy database and Hotpep search against the conserved CAZyme short peptide sequence database, the classification of *gt*s was considered valid if consistent across at least two tools [36]. Prediction of the transmembrane domains was carried out using TMHMM Server v. 2.0 [37].

All-against-all bi-directional BLASTP was carried out on the whole set of putative proteins with cut-off of at least 50% identity and 50% query coverage. The blast output was used to group the proteins based on their function using Markov clustering (MCL) in the mclblastine v12-0678 pipeline [38]. Further hierarchical clustering was computed in TM4 MeV Suite, version 4.9 based on the presence/absence of the protein families in the EPS clusters [39]. An HCL tree was visualized in Interactive Tree of life [40] by importing a Newick tree from TM4 MeV Suite [39].

## 3. Results and Discussion

### 3.1. Number of Clusters and Gene Composition

With the aim of understanding the diversity in the gene clusters encoding the proteins required for EPS biosynthesis in several lactobacilli genomes, we identified EPS gene clusters in 100 sequenced *Lactobacillus* genomes. Further, their gene composition and diversity in the putative proteins encoded by these genes were analyzed by a homology-based approach. *Lactobacillus* species are broadly classified into three main lifestyles, viz., host-adapted, nomadic and free-living based on factors such as frequency of isolation from specific sources, metabolic characteristics and stress resistance [35]. The genomic features of lactobacilli such as genome size, GC content and the presence and absence of certain genes have been shown to be correlated to these habitats [35]. Considering this and the established physiological roles of the EPS in the environmental interactions, it was hypothesized that gene composition of the EPS clusters is correlated with the above-mentioned lifestyles. To test this, an attempt was also made to analyze the correlation of the organization of an EPS cluster as well as the gene composition of the clusters with these lifestyles. These results are discussed in the following sections wherever relevant.

A total of 146 EPS gene clusters were detected in the genomes of 100 of the total 106 *Lactobacillus* strains examined belonging to 27 species (Figure S1, Tables S1 and S2). Only three EPS gene clusters from *L. plantarum* 16, *L. buchneri* CD034 and *L. buchneri* NRRL B-30929 were found to be located on the plasmids, whereas the rest were encoded by the chromosomal genome. The number of clusters in the strains were one (65 strains), two (25 strains), three (9 strains) or four (1 strain). EPS biosynthesis, transport and regulation in LAB have been shown to require a set of a few essential genes, which are usually present within the EPS clusters [24]. The essential genes in the clusters include priming glycosyltransferase (*epsE*), glycosyltransferase (*gt*), flippase (*wzx*), polysaccharide polymerase (*wzy*), tyrosine kinase (*epsC*) and tyrosine kinase modulator (*epsB*). Other genes which are often present as a part of some of the EPS clusters but which are either considered to be dispensable or were reported earlier to be present elsewhere in the genome include LytR transcriptional regulator (*epsA*), phosphotyrosine phosphatase (*epsD*), genes involved in the generation of activated sugar precursors and acetyl- and pyruvyl transferase involved in the chemical decoration of the EPS [24]. Detailed analysis of only the essential genes as mentioned above was further conducted.

The GC contents of *gt*, *wzx* and *wzy* were relatively lower whereas those of *epsA*, *B*, *C*, *D* and *E* were similar to the whole genome GC content (Figure S2). This is consistent with earlier studies in *Streptococcus pneumoniae* [41], wherein it was shown that the GC content of *epsA*, *B*, *C* and *D* was similar to that of the whole genome and that of *wzx* and *wzy* was much lower. Several EPS clusters were also found to have transposable elements. Amongst all the genes, the highest proportion of *wzx* genes (9.5%) had the transpose element present within or adjoining them. These proportions were 3.7–6.7% for *epsE*, *gt*, *wzy* and the precursor biosynthesis genes and 1% for *epsD*. This observation along with the lower GC content suggests that at least *wzx*, *wzy* and *gt* might have been acquired by horizontal gene transfer (HGT). This speculation is also in agreement with the earlier studies on *S. pneumoniae*, *L. delbreuckii* and *S. thermophilus*, as well as in gram-negative bacteria [41,42].

Some of the clusters did not have all the above-mentioned genes which are essential for EPS biosynthesis. In such incomplete clusters, polysaccharide polymerase (*wzy*) was the most common missing gene or pseudogene (absent in 42 clusters), followed by phosphoregulatory module (*epsB* and *C* or both, absent in 33 clusters), flippase (*wzx*, absent in 18 clusters) and priming glycosyltransferase (*epsE*, absent in 10 clusters) (Table 1). In the strains having multiple clusters, such lack of the crucial genes is likely to be compensated by the genes from the other clusters. Such dependency of the EPS clusters on one another was demonstrated in *L. plantarum* WCFS1. Deletion of one cluster caused reduction in the molecular weight of the EPS, whereas knockout of others resulted either in changed monosaccharide composition or reduced EPS yield [31]. Notably, two of these four clusters in *L. plantarum* WCFS1 were incomplete. In the present study, some of strains having incomplete clusters also had multiple clusters. Thus, it is possible that this incompleteness in some clusters is complemented by the other clusters in the same strain.

### 3.2. Organization of Genes in the Clusters

**Figure 1:**
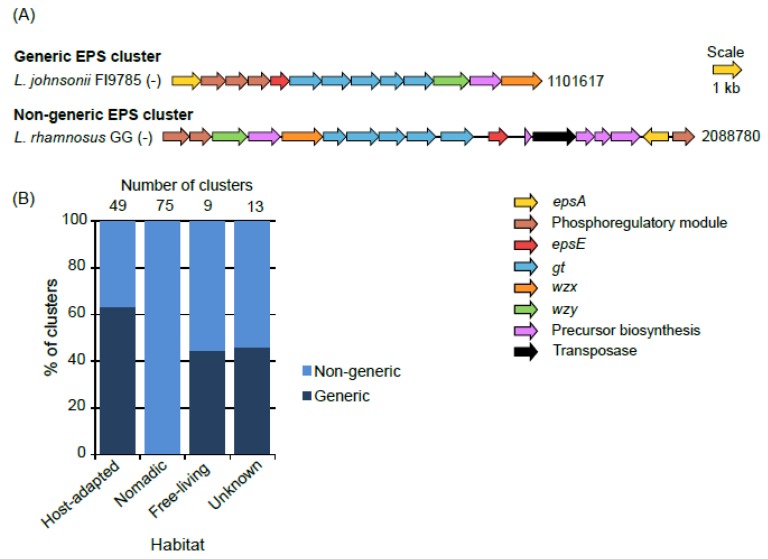
Examples of generic and non-generic EPS gene clusters (**A**) and their proportions in various habitat groups (**B**). Numbers at the end of the gene clusters in (**A**) denote the position of the terminal nucleotide on that side of the cluster as per the annotated genome sequences in the NCBI genome database. A negative sign in parentheses at the end of strain names indicates that the clusters were encoded by the negative strand.

In the most common form of organization of the genes in the EPS clusters, a stretch of the first five genes on the 5’end, *epsABCDE*, is highly conserved and such organization has been referred to as “generic” [24,43,44]. In our study, we found many clusters wherein *epsABCDE* stretch was absent, either because of absence of some genes from this stretch or placement of the one or more of these five genes somewhere else in the EPS cluster. We named such clusters “non-generic”. In this way, only 41 of the total 146 clusters were found to be generic (Figure 1). None of the clusters from the nomadic were generic which was because of the complete absence of *epsA* in them. On the other hand, a large proportion (65%) of the clusters from host-adapted group was generic.

**Figure 2:**
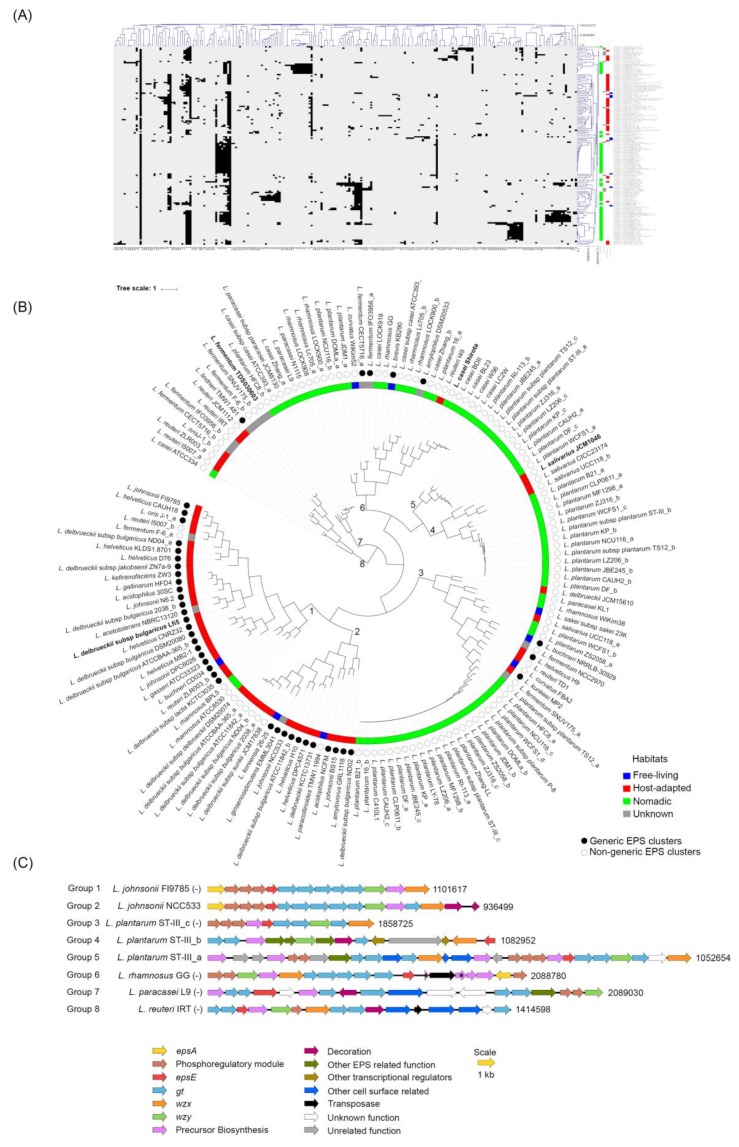
HCL analysis based on the presence and absence of the protein families across the EPS gene clusters identified in the *Lactobacillus* genomes indicating (**A**) a heat map wherein black and white areas represent presence and absence, respectively, of the protein families, (**B**) grouping of the EPS cluster based on the HCL analysis and (**C**) representative EPS clusters from each of the eight groups identified in (**B**). Alphabets after the strain names denote multiple clusters found in some strains. Pseudogenes and the genes which encode for the truncated proteins and hence are likely to be non-functional are indicated by asterisks. Description for numbers at the ends of the clusters and of negative signs in parentheses is given in the legend of Figure 1.

In general, in the generic clusters, the *epsABCDE* region was followed by several *gt* genes and subsequently by *wzx* and *wzy* (Figure 1). The pattern of organization of genes within each non-generic cluster appeared to be mosaic and was also highly variable amongst these non-generic clusters. The only common factor in all the clusters was that, wherever present, *epsB* and *C* were always in tandem with each other (Table S2). Considering such high variability observed in the EPS clusters across the strains and to get insights into the similarity of the EPS clusters with each other in terms of the gene content, all-against-all BLASTP was performed for all the coding sequences present in the EPS gene clusters followed by Markov clustering (MCL) analysis which depicted the presence of 233 families representing all the genes in the EPS gene clusters (data not shown). This analysis showed that some protein families were common across many EPS clusters, whereas a few others were specific to certain EPS clusters (Figure 2A). HCL analysis showed the presence of eight distinct groups of EPS clusters in the tree (Figure 2B,C). The majority of the EPS clusters from the host-adapted and nomadic habitats were found in the mutually exclusive groups, 1 to 2 and 3 to 7, respectively. On the other hand, EPS clusters from the free-living habitats, in spite of being small in numbers (8) were found across four groups, viz., 1, 2, 3 and 6, which were shared with the clusters from host-adapted and nomadic strains. These observations suggest the EPS clusters from the host-adapted and nomadic habitats are distinct from each other in terms of the gene content, whereas some of the clusters from the free living strains are similar to host adapted, while few others are similar to the nomadic strains. This speculation is consistent with the fact that free-living lactobacilli are considered to be ancestral and are phyletically broadly distributed, whereas host-adapted and nomadic species are considered to have evolved from the free-living ancestors [35]. *L. plantarum* represented the most diverse species in terms of having its EPS clusters present across the highest number of groups (five groups, 3 to 7). This finding is consistent with the earlier report stating that EPS clusters are the most varied regions amongst the genomes of several *L. plantarum* strains [33].

### 3.3. Variation in the Number of Protein Families across Various Gene Functionalities

To get insights into the variations in each of the essential genes in the EPS clusters across various EPS gene clusters, families of the putative proteins encoded by them were analyzed by the PATtyFams approach [45]. In this approach, functions are assigned to the putative proteins encoded by genes based on the k-mer signatures [45]. Within a genus, proteins with similar functions are pooled into a single local genus-level family (PLFam) and similar pooling across genera gives rise to the global families (PGFams). Thus, PLFams represent subtypes of PGFams and both have been used to get insights into diversity in the putative proteins encoded by bacterial genomes [46,47,48,49]. The classification of the putative proteins encoded by the identified EPS clusters into these families already available at PATRIC database was used to decipher the closeness of the homologous genes.

GT, Wzx and Wzy had the highest number of PLFams as well as PGFams, the highest proportion of singleton families and the lowest average number of proteins per family (Table 1). On the other hand, EpsA, EpsD and EpsE had the lowest total number of families as well as proportion of singleton families and highest number of proteins per family. Similar values were observed for EpsB and C. These trends indicate low variation in EpsA, B, C, D and E and high diversity in GT, Wzx and Wzy across *Lactobacillus* EPS clusters. These observations are consistent with earlier reports in which it has been shown that genes encoding EpsA, B, C, D and E are conserved not only in *Lactobacillus*, but across various LAB genera [24]. The high degree of variation observed in GT, Wzx and Wzy is also consistent with the studies on gram-positive bacteria such as *S. pneuminae* and *Oenococcus oeni* and gram-negative bacteria such as *Acinetobacter*, *Salmonella* and *Yersinia* [41,50,51]. Indeed, in many of these bacteria, GT, Wzx and Wzy were found to be serotype-specific proteins in contrast to the other proteins encoded by the polysaccharide biosynthesis clusters.

Some clusters also had more than one copy of some genes except *epsD* which was always present in one copy or absent in some clusters (Table 1). Most of such multi-copy genes within a cluster, except *epsA*, belonged to the different protein families as indicated by the ratio of number of such multicopy gene within a cluster: protein family, which was in the range of 1-1.3 (Table 1). This possibly indicates non-redundant function of the multi-copy genes in EPS gene clusters. In the following sections, we discuss unique results observed for each of the putative proteins encoded by the EPS clusters.

### 3.4. EpsA

Two largest PLFams of EpsA (PLF_1578_00001102 and PLF_1578_00003813) were also most widely distributed across highest number of species (Table S2). EPS clusters of all the strains of any given species had gene encoding EpsA belonging to the same PLFam, except *L. delbreuckii*, in which two families were found. None of the EPS clusters in *L. plantarum* had *epsA* associated with them. Highly diverse molecular functions have till now been ascribed to EpsA. In several gram-positive bacteria, EpsA has been shown to be required for the attachment of capsular polysaccharides (CPS) to the cell wall [52,53]. In a few others, it has been shown as a positive regulator of EPS biosynthesis [54,55,56] and in some cases as a transcriptional attenuator [57]. The presence of *epsA* has been shown to be highly essential for EPS production in *L. johnsonii* [58]. On the other hand, in *S. pneumoniae*, the deletion of *cps2A* (similar to *Lactobacillus epsA*) caused only the lowering of CPS production [59]. Considering this, the lack of *epsA* in *L. plantarum* clusters remains intriguing. In *L. plantarum* WCFS1, a related ORF, lp_1000, similar to *epsA* was present at a distant location from EPS clusters and has been shown to be involved in biofilm formation [60,61]. It is possible that the protein encoded by this gene might function as EpsA in *L. plantarum*.

### 3.5. Phosphoregulatory Module: EpsB, C and D

Of the 146 clusters, 95 contained all the three genes (*epsB*, *C* and *D*) of the phosphoregulatory module, 21 lacked all three genes, 17 lacked only *epsD*, 2 lacked only *epsC* and 11 clusters contained only one of these three genes. Absence of *epsD* across relatively larger proportion of EPS gene clusters (Table 1) supports the earlier observation in *S. thermophilus* that *epsD* is dispensable for EPS biosynthesis [62]. It was also shown that *epsD* mutants of *Bacillus subtilis* had similar EPS production as that of wild-type and the phosphorylated state of the tyrosine kinase (*epsC*) was considered to be regulated by proteolysis rather than dephosphorylation [63]. Furthermore, the presence of only one PGFam and a very low number of PLFam across all the analyzed *Lactobacillus* EPS clusters, the presence of only one PLFam in each species except *L. plantarum* and the absence of multiple copies of *epsD* in each cluster points towards highly conserved nature of *epsD* across *Lactobacillus* EPS clusters analyzed in the present study.

**Figure 3:**
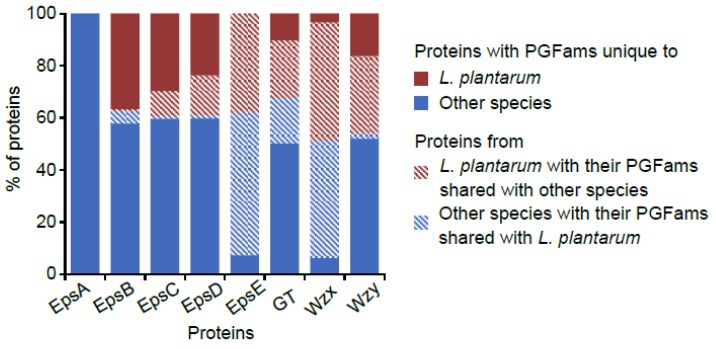
Proportions of various proteins for which PGFams were shared between and unique to *L. plantarum* and other species.

For EpsB and EpsC, the highest numbers of PLFams as well as the biggest PLFams (PLF_1578_00005921 and PLF_1578_00008784, respectively) were found in *L. plantarum* (Table S2) However, the most widely distributed PLFams of EpsB and EpsC (PLF_1578_00003923 and PLF_1578_00002999, respectively) (found across several species) did not have their members in *L. plantarum*. Similarly, PGFams of the majority of the genes of EpsB, C and D were mutually exclusive between *L. plantarum* and other species (Figure 3). This fact probably suggests a unique nature of the phosporegulatory module and the associated mechanism of EPS regulation in *L. plantarum*. Based on some of earlier studies, EpsA and the phosphoregulatory proteins appear to modulate each other’s activities. In *S. agalactiae*, CpsC (similar to *Lactobacillus* EpsB) was shown to physically interact with and regulate the activity of CpsA (similar to *Lactobacillus* EpsA) of attaching the polysaccharide to the cell wall [64]. Furthermore, in *S. pneumoniae*, a decreased level of tyrosine-phosphorylated Cps2D (similar to *Lactobacillus* EpsC) was observed upon deletion of *cps2A* [59]. These observations indicate that physical crosstalks of EpsA with the phosphoregulatory module are possible in *Lactobacillus* also. This might explain the unique nature of EpsB and C in *L. plantarum*, wherein *epsA* was completely absent from the EPS clusters.

### 3.6. EpsE

*L. plantarum*, *L. casei* and *L. fermentum* were the most diverse species in terms of having the highest number of PLFams of EpsE. While the majority of the EpsE had the size 209–241 amino acids, a different type of EpsE having 466 amino acids and belonging to PLF_1578_00002511 was found in 10 clusters from *L. paracasei*, *L. rhamnosus* and *L. casei* (Table S2). EpsE of the longer length (455 amino acids) has also been reported earlier in *S. salivarius* [65]. However, this protein showed very low (<30%) sequence identity with the above-mentioned *Lactobacillus* EpsE (data not shown).

Very few EpsE proteins have been functionally characterized in *Lactobacillus*. EpsE from *L. rhamnosus* GG has been shown to be a galactosyl-1-P transferase [6]. In the present study, this protein was found to belong to the PLF_1578_00034667, which was the fourth largest and the most widespread PLFam with 14 members found across eight species (Table S2). Similarly, EpsE from *L. johnsonii* FI9785 has been characterized as a galactosyltransferase [8]. We found that this protein belonged to PLF_1578_00003576, which was the second largest as well as the second most widely distributed PLFam. Only one EpsE has till now been characterized as a glucosyltransferase in *Lactobacillus* [26]. In the current dataset, PLFam of this EpsE was the fifth largest with 10 members of which nine were restricted to *L. delbreuckii*. Based on these observations, it can be surmised that priming galactosyltransferase might be dominant over the glucosyltransferase in *Lactobacillus* EPS clusters. However, EpsE proteins with considerable sequence homology were shown to have different substrate specificities [24], suggesting that the functional characterization of diverse EpsE in *Lactobacillus* would be required to conclude anything about the substrate specificity determining factors.

### 3.7. GTs

**Figure 4:**
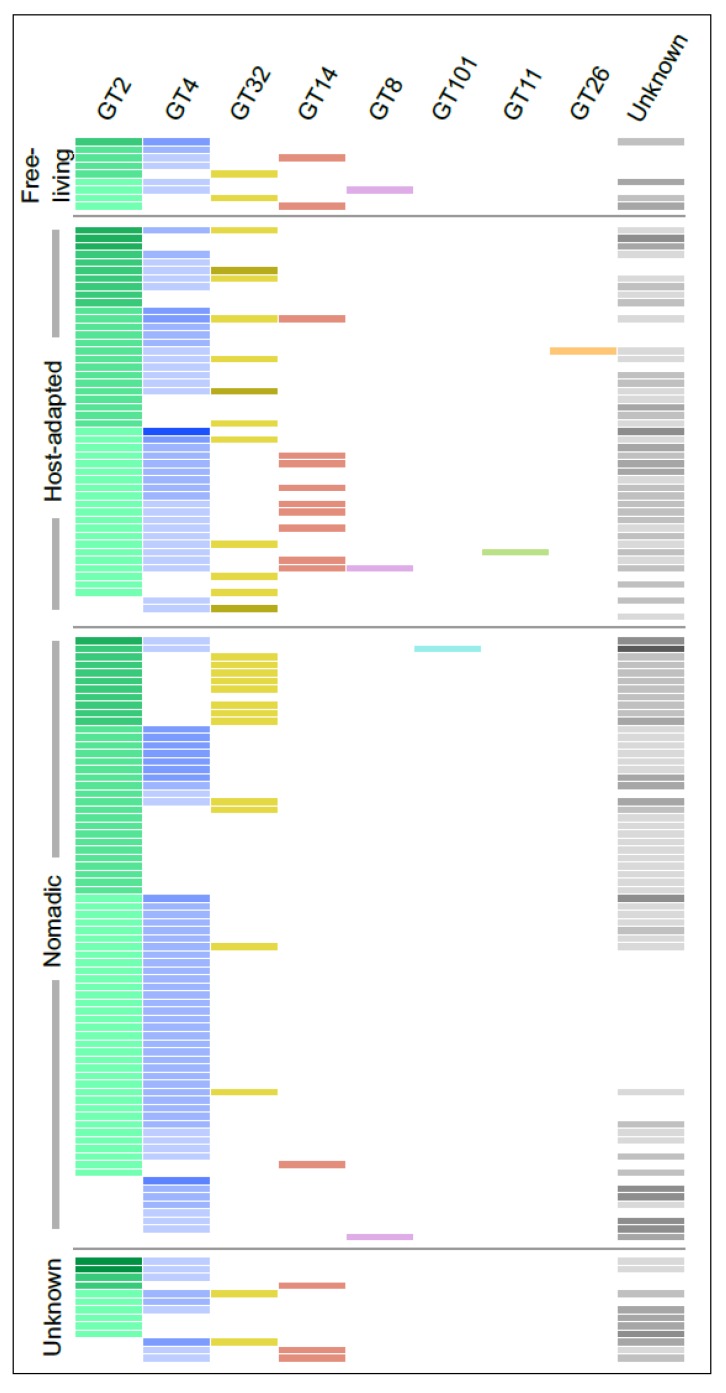
The distribution of various families of GTs encoded by the *Lactobacillus* EPS gene clusters identified using the dbCAN2 web server [36]. Each row represents a single EPS gene cluster and each column a family. The lightest shade across the columns indicates the presence of a single member of that family in that cluster while darker shades denote multiple members. The unknown column indicates GTs for which no information could be obtained using dbCAN2 database.

GTs represented the largest and most diverse group of proteins encoded by the *Lactobacillus* EPS gene clusters and belonged to numerous PLFams, many of which were singletons. Considering this, we used the dbCAN2 server, which classifies GTs into several classes based on amino acid sequence similarity linked to the specificity of enzyme and its 3D structure information [66]. In this way, of the 670 GTs, 469 could be classified into 8 CAZy families with number of members ranging from 1 to 232, whereas 200 could not be annotated to any family. GT2 and GT4 were the largest families accounting together for more than 60% of all the GT proteins. Interestingly, GT14 and GT32 were mutually exclusive in all the clusters except the one found in *L. salivarius* UCC118 (Figure 4). Furthermore, genes for the GT14 family were absent in all-but-one clusters from the nomadic group. It is challenging to speculate on the reasons for this mutual exclusion as only one GT32 has been functionally characterized in gram-positive bacteria [67], whereas no bacterial GT14 have been characterized till date to the best of our knowledge. The characterized GT32 from *S. pneumoniae* was shown to transfer α-N-acetylglucosamine as well as α-glucose [67]. On the other hand, enzymes belonging to GT14 from the other organisms are of an inverting type with N-acetylglucosamine as one of the most common sugars transferred by both the families [66]. Based on these observations, it is tempting to speculate that *Lactobacillus* EPS with N-acetylglucosamine can have it either in α or β linkage but not both. However, GTs are known to be highly promiscuous in nature [24], which in addition to very scarce studies on their functional characterization in LAB makes it difficult to predict their substrate specificity.

### 3.8. Wzx and Wzy

**Figure 5:**
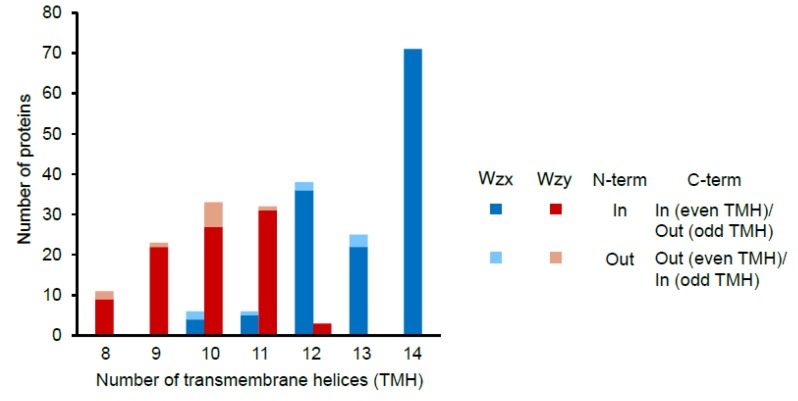
Number of transmembrane helices predicted using the TMHMM server v. 2.0 [37] to be present in the putative Wzx and Wzy proteins encoded by the *Lactobacillus* EPS gene clusters.

Seventeen EPS clusters contained multiple copies of genes encoding Wzx (Table 1). This was the second highest number of multi-copy genes after *gt*. Sixteen of these clusters belonged to *L. plantarum* and half of them also had multiple copies of phosphoregulatory module, *gt* and precursor biosynthesis genes. This probably suggests that such clusters might be responsible for the biosynthesis of two types of EPS. All Wzx and Wzy proteins were predicted to be transmembrane proteins with 10 to 14 (mode 14) and 8 to 12 (mode 10) transmembrane helices, respectively (Figure 5). These numbers are in slight disagreement with the earlier reported numbers of 12 and 10–14 for *Pseudomonas* and other gram-negative bacteria [68] pointing towards the possibly unique nature of the *Lactobacillus* Wzx and Wzy. For more than 90% of both Wzx and Wzy proteins, N-terminal was predicted to be present inside the cytoplasm. Of these, the majority of Wzx and Wzy had their C-terminals inside and outside of the cytoplasm, respectively. A lack in the knowledge about the structure–function relationships of the Wzx and Wzy with the EPS biosynthesis in gram-positive bacteria along with high variation in these proteins within *Lactobacillus* limits our scope of concluding anything about these observations. No correlation of the number of transmembrane helices in Wzx and Wzy with the habitats or type of clusters (generic or non-generic) was observed (data not shown).

### 3.9. Precursor Biosynthesis

**Figure 6:**
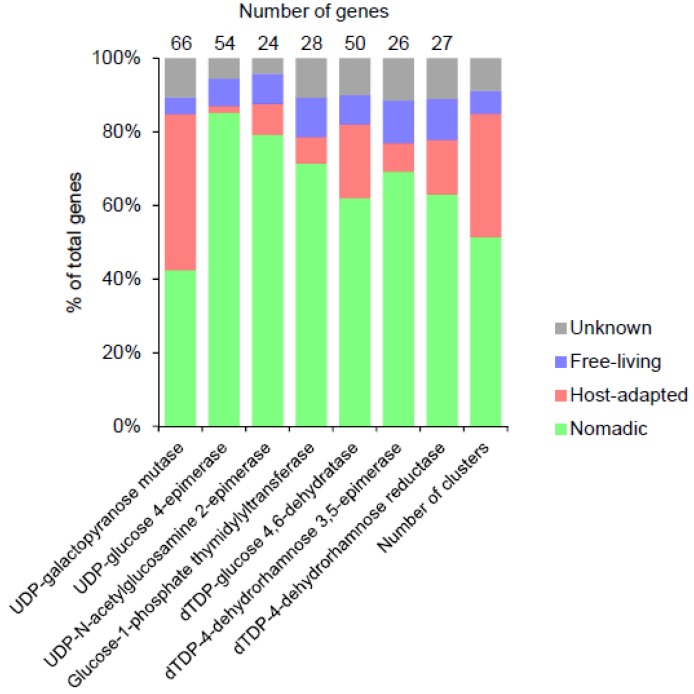
The distribution of the most abundant sugar nucleotide precursor biosynthesis genes in the EPS gene clusters in *Lactobacillus* species from various habitats. Other precursor biosynthesis genes detected in the clusters (and their numbers) were UTP-glucose-1-phosphate uridylyltransferase (6), UDP-glucose 6-dehydrogenase (3), UDP-N-acetylglucosamine 4-epimerase (2), UDP-phosphate galactose phosphotransferase (1), galactofuransyltransferase (1), mannose-1-phosphate guanylyltransferase (1), UDP-N-acetylglucosamine 4,6-dehydratase (1), phosphomannomutase (1), 4-keto-6-deoxy-N-acetyl-D-hexosaminyl-(lipid carrier) aminotransferase (1), UDP-glucuronate 4-epimerase (1) and UDP-N-acetylgalactosaminyltransferase (1).

Some of the clusters also contained genes involved in the biosynthesis of the nucleotide sugar precursors. Within this category, UDP-galactopyranose mutase was the most common gene found across 66 EPS gene cluster (Figure 6). This is consistent with the occurrence of galactofuranose in many *Lactobacillus* EPS [13,24,69].

UDP-glucose 4-epimerase (GalE) was the next most abundant precursor biosynthesis gene found across 54 EPS clusters. GalE has been shown to interconvert either hexoses (glucose/galactose) or N-acetylhexosamines (N-acetylglucosamine/N-acetylgalactosamine) or both [70]. GalE from *L. plantarum* WCFS1 (NP_784866) belongs to the phylogenetic cluster of GalE which prefers N-acetylhexosamine as the substrate [70]. We found that NP_784866 belongs to one (PLF_1578_00057321) of the two largest families of GalE which had almost all its members limited to *L. plantarum* EPS clusters. Thus, the presence of N-acetylglucosamine in the *L. plantarum* WCFS1 EPS [31], which is likely because of NP_784866, is possibly conserved across other *L. plantarum* EPS. While GalE was encoded by only one EPS cluster from the host-adapted group, the product of the similar enzyme encoded by the housekeeping gene can be used for the incorporation of the corresponding sugar in EPS (see below).

Twenty-four clusters contained a set of all four genes usually referred to as *rmlA*, *B*, *C* and *D* required for the biosynthesis of dTDP-L-rhamnose [71]. These genes encode for glucose-1-phosphate thymidylyltransferase, dTDP-glucose 4,6-dehydratase, dTDP-4-dehydrorhamnose 3,5-epimerase and dTDP-4-dehydrorhamnose reductase, respectively. In some cases, *rmlABCD* operon was not present within the EPS cluster but downstream from the cluster after a gap of a few unrelated genes (data not shown). Indeed, it is well known that the genes outside EPS clusters also contribute to the biosynthesis of activated nucleotide sugar precursors. Six (including N-acetylgalactosamine, galactose and rhamnose) of about 11 sugars commonly reported in LAB EPS have been estimated to be supplied by the housekeeping pathways [24]. Thus, while the presence of certain precursor biosynthesis genes in the EPS cluster indicates the presence of that sugar in the EPS, absence of the genes cannot be taken as an indicator of absence of that sugar in the EPS.

Genes encoding UDP-N-acetylglucosamine-2-epimerase, which is responsible for the presence of N-acetyl-mannosamine or N-acetyl-mannosaminuronic acid in the EPS, was found across 24 EPS clusters. This was an unexpected finding, as very few strains of *Lactobacillus* have till now been shown to have these sugars as a constituent of their EPS [24,50].

### 3.10. Other Genes

Some of the clusters from *L. plantarum,* including WCFS1, belonging to groups 4, 5 and 6 in the MCL tree (Figure 2) also had other types of transcriptional regulators annotated to belong to MarR and AraC families (Table S1). In *L. plantarum* WCFS1, the MarR family transcription factor encoded by lp_1230 has been proposed to be involved in the transcription of the immediately upstream gene, mannose-specific adhesin [72], which was also present in many other EPS clusters. Notably, in *Sinorhizobium meliloti*, *ExpG,* which is another MarR family transcriptional regulator, as well as an AraC family transcriptional regulator, has been shown to be involved in the production of EPS [73,74]. In light of this fact, the possibility of the potential involvement of MarR and AraC family transcription factors in EPS production by *L. plantarum* strains remains to be explored.

Some of the clusters also had genes that have been annotated to encode for polysaccharide biosynthesis proteins, lipopolysaccharide biosynthesis protein and hypothetical proteins. These genes showed no or very low similarity to the core genes required for EPS biosynthesis (data not shown); nevertheless, they might have some uncharacterized function in the EPS biosynthesis. As a subset of these genes also had several transmembrane helices, it is possible that they might encode for novel Wzx and Wzy and can compensate for the missing well-annotated *wzx* and *wzy* in some such clusters. Apart from *Lactobacillus*, the presence of such genes, for which the functions in EPS biosynthesis are not clearly annotated, has also been reported in EPS gene clusters of *O. oeni* [50].

### 3.11. Sharing of Protein Families Across Various Habitats

**Figure 7:**
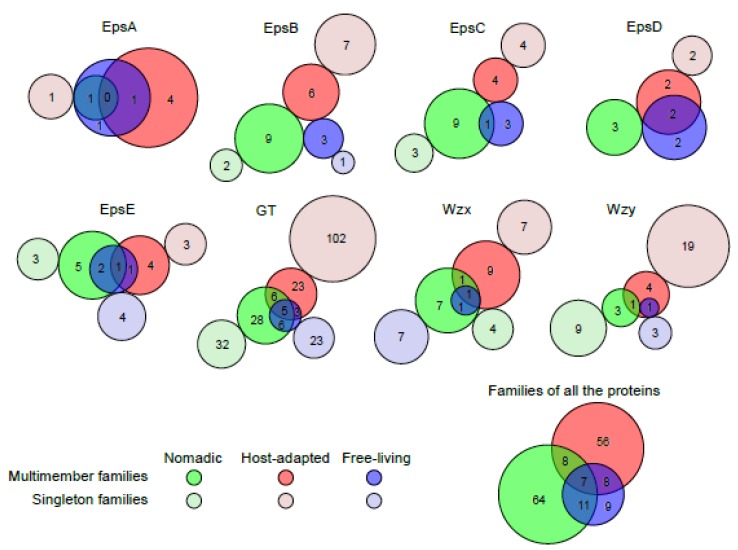
The sharing of PATtyFams families of various proteins encoded by the EPS gene clusters in *Lactobacillus* species from various habitats. PLFams were considered for all the proteins except GT and Wzy for which PGFams were analyzed. Families and gene clusters from unknown habitats were excluded. Area-proportional Venn diagrams were drawn for families with more than one member using BioVenn program [75] and area-proportional circles were manually added for the singleton families.

To understand if the distribution of the families of the proteins (PLFams and PGFams) encoded by EPS clusters in *Lactobacillus* is dependent on the habitat in which these strains are found, grouping of the protein families according to the habitats was analyzed. EpsA was found in very few clusters from nomadic groups and the only family found in nomadic groups was shared with free-living groups. For EpsB, C and D, families from host-adapted and nomadic groups were completely mutually exclusive, whereas one and two families of EpsC and EpsD, each from free-living groups, were shared with nomadic and host-adapted groups, respectively. For EpsE, GT, Wzx and Wzy, many families in the host-adapted and nomadic groups were mutually exclusive to one another (Figure 7). However, no family with multiple members was unique to the free-living group. Taken together, the least sharing of protein families was observed between host-adapted and nomadic strains, whereas families from free-living strains were highly shared with two other habitats. This observation is consistent with the MCL analysis showing distinct grouping in the EPS clusters from host-adapted and nomadic habitats and supports the postulated ancestral nature of lactobacilli from the free-living habitat [35].

In *B. subtilis*, EpsA and B (homologues of *Lactobacillus* EpsB and C, respectively) have been shown to function in auto-regulation of the EPS production and this phenomenon has been postulated to be conserved across other bacteria as well [63]. Since EPS appear to have many roles in the environmental interactions in lactobacilli, the presence of habitat-specific families, at least in the case of EpsB, C and D, suggests that the mechanisms of such auto-regulation of the EPS biosynthesis mediated by these genes might be habitat-specific. Host-adapted lactobacilli live under very high bacterial cell densities in the vertebrate intestine and also have reduced genome sizes because of the nutrient-rich environment [35,76]. The nomadic lactobacilli, however, can be found under diverse environments and thus have higher metabolic flexibility [33,35]. Such varying environmental conditions thus might demand different ways of regulation of EPS via the phosphoregulatory module, which can account for the distinct natures of EpsB, C and D in nomadic and host-adapted habitats. While many habitat-specific families of other proteins such as EpsE, GT, Wzx and Wzy which decide the composition of EPS were found, no obvious differences in the EPS composition of the lactobacilli belonging to different niches has been reported. This could be justified by the fact that in *B. subtilis*, at least EpsE is further regulated by phosphorylation mediated by EpsA and B [63]. Thus, habitat-specific families of these proteins might not govern the habitat-specific EPS composition but might be involved in regulating the EPS biosynthesis based on the environment-specific signals.

## 4. Conclusions

In summary, this study highlights the immense diversity in the EPS biosynthesis gene clusters in *Lactobacillus*. Some of the striking observations regarding habitat-wise properties of the EPS clusters and genes need further investigation. These include the absence of *epsA* in nomadic strains, mosaic arrangement of genes in many clusters and the mutual exclusion of the families of many proteins such as EpsA, B and C and GT between host-adapted and nomadic habitats. The much higher variation observed in GT, Wzx and Wzy further demands undertaking a humongous task of functional characterization of this massively diverse pool of enzymes and correlating their properties with EPS biosynthesis and regulation.

## Figures and Tables

**Table 1:** Details of the essential genes in the *Lactobacillus* EPS gene clusters and families of the encoded putative proteins.

	Genes	Abbreviation	Total Number of Genes	Number of PLFams	Number of Clusters not Having the Gene	Number of PGFams	Proteins: PLFams	PLFams: PGFams	% of Singleton Families #	Number of Clusters Having Multicopy Genes	For Clusters Having >2 Copies of Gene, Average Number of Those	
											Genes	Families #
1	LytR-transcriptional regulator	*epsA*	78	8	74	2	9.8	4	12.5	5	2.2	1
2	Tyrosine kinase modulator	*epsB*	130	29	25	15	4.4	1.9	37.9	9	2	2
3	Tyrosine kinase	*epsC*	125	25	30	10	5	2.5	28	8	2	2
4	Phosphotyrosine phosphatase	*epsD*	97	11	49	1	8.8	11	9.1	0	-	-
5	Priming glycosyltransferase	*epsE*	140	24	10	8	5.8	3	33.3	4	2	1.8
6	Glycosyltransferase	*gt*	670	343	0	246	1.9	1.4	66.6	140	4.6	4.3
7	Flippase	*wzx*	147	39	18	16	3.8	2.4	46.2	17	2.17	2
8	Polysaccharide polymerase	*wzy*	103	50	42	42	2	1.2	73.8	2	2	2

# PGFams were considered for GT and Wzy, while PLFams were considered for all other proteins.

## Supplementary Materials

The following are available online at https://www.mdpi.com/2076-2607/7/10/444/s1, Figure S1: Organization of the 146 EPS gene clusters identified in 100 *Lactobacillus* strains. Group numbers indicate groups of the clusters obtained in the HCL analysis (Figure 2). Numbers at the end of the clusters denote the position of the terminal nucleotide on that side of the cluster as per the annotated genome sequences in NCBI. Alphabets after the strain names denote multiple clusters found in some strains. Negative sign in parenthesis at the end of strain names indicates that those clusters were encoded by the negative strand. Pseudogenes and the genes which encode for the truncated proteins and hence are likely to be non-functional are indicated by asterisks. Names of representative EPS clusters from each group shown in Figure 2 are indicated in boldface. Figure S2: GC content of the genes detected in the EPS clusters identified in the sequenced *Lactobacillus* genomes. Numbers on the top indicate the average GC content for that gene. Table S1: List of the *Lactobacillus* strains analyzed in the present study.; Table S2: Details of the ORFs detected in the EPS gene clusters identified in *Lactobacillus* genomes.
