# Supplementary figures and images for: A Diverse Repertoire of Exopolysaccharide Biosynthesis Gene Clusters in Lactobacillus Revealed by Comparative Analysis in 106 Sequenced Genomes
Source: Microorganisms. 2019 Oct 11;7(10):444. doi: 10.3390/microorganisms7100444 (PMC6843789; doi:10.3390/microorganisms7100444)

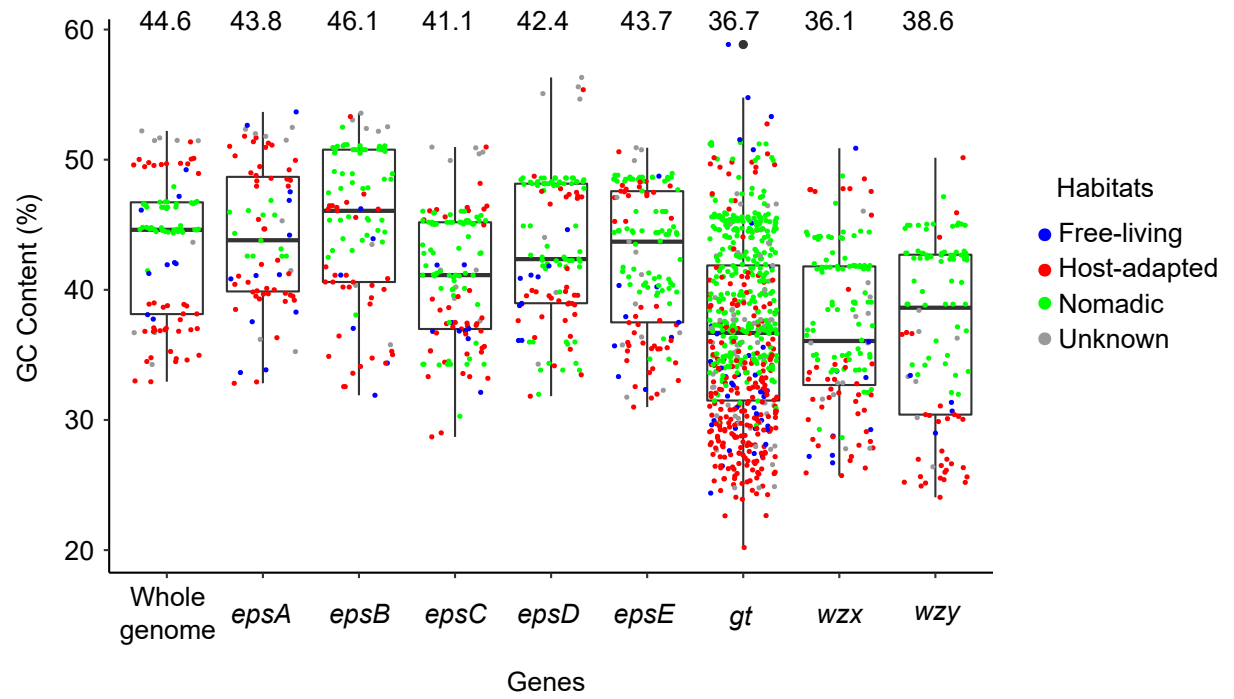

Supplement: Supplementary file 1 [file microorganisms-07-00444-s001.zip › FigureS2.pdf]
