# Supplementary material for: A Diverse Repertoire of Exopolysaccharide Biosynthesis Gene Clusters in Lactobacillus Revealed by Comparative Analysis in 106 Sequenced Genomes
Source: Microorganisms. 2019 Oct 11;7(10):444. doi: 10.3390/microorganisms7100444 (PMC6843789; doi:10.3390/microorganisms7100444)
Supplement: Supplementary file 1 [file FigureS1.pdf › FigureS1.pdf]

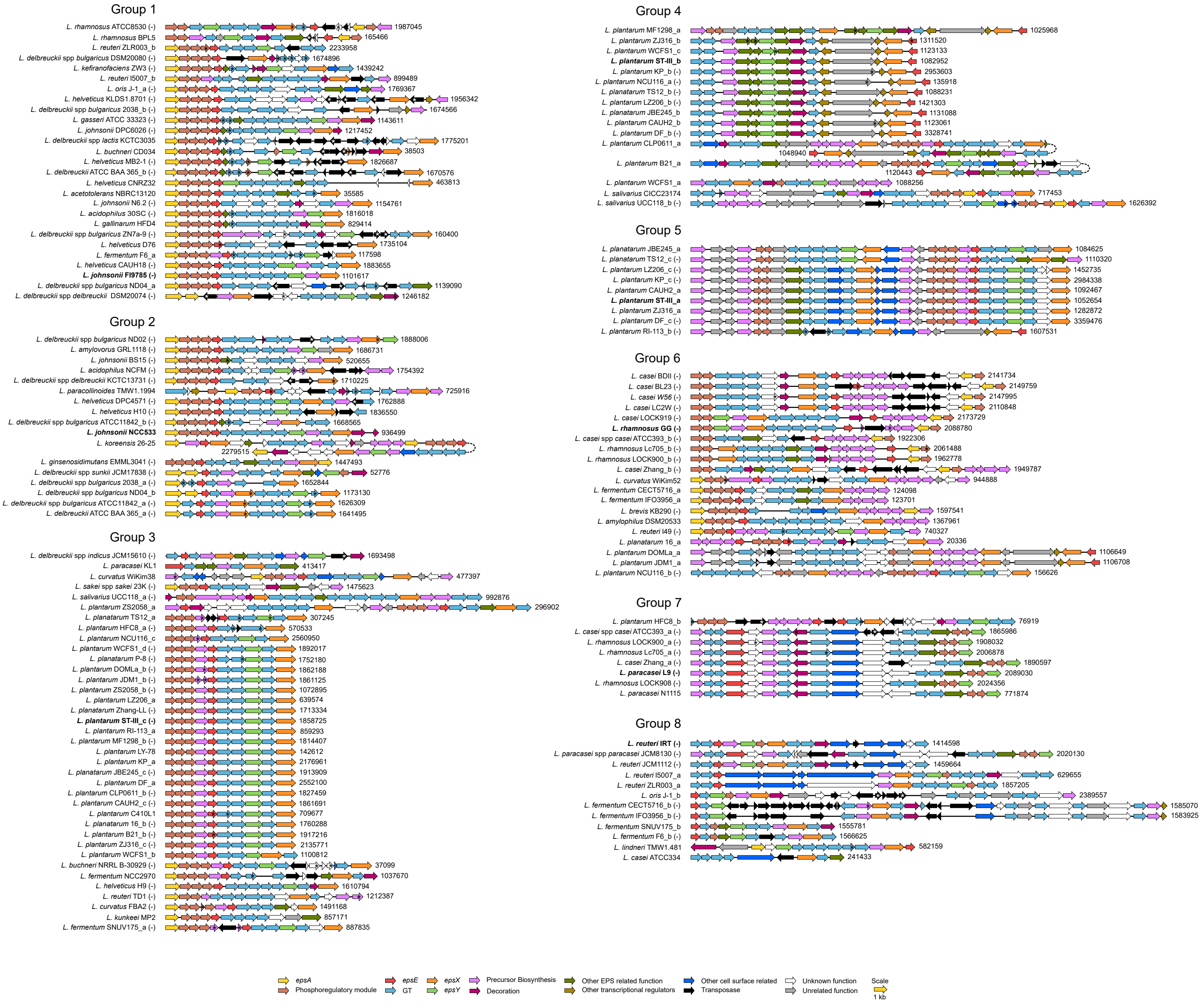
Group 1
L. rhamnosus ATCC8530 (-) 1987045
L. rhamnosus BPL5 165466
L. reuteri ZLR003_b 2233958
L. delbrueckii spp bulgaricus DSM20080 (-) 1674896
L. kefiranofaciens ZW3 (-) 1439242
L. reuteri I5007_b 899489
L. oris J-1_a (-) 1769367
L. helveticus KLDS1.8701 (-) 1956342
L. delbrueckii spp bulgaricus 2038_b (-) 1674566
L. gasseri ATCC 33323 (-) 1143611
L. johnsonii DPC6026 (-) 1217452
L. delbrueckii spp lactis KCTC3035 1775201
L. buchneri CD034 38503
L. helveticus MB2-1 (-) 1826687
L. delbrueckii ATCC BAA 365_b (-) 1670576
L. helveticus CNRZ32 463813
L. acetotolerans NBRC13120 35585
L. johnsonii N6.2 (-) 1154761
L. acidophilus 30SC (-) 1816018
L. gallinarum HFD4 829414
L. delbrueckii spp bulgaricus ZN7a-9 (-) 160400
L. helveticus D76 1735104
L. fermentum F6_a 117598
L. helveticus CAUH18 (-) 1883655
L. johnsonii FI9785 (-) 1101617
L. delbrueckii spp bulgaricus ND04_a 1139090
L. delbrueckii spp delbrueckii DSM20074 (-) 1246182
Group 2
L. delbrueckii spp bulgaricus ND02 (-) 1888006
L. amylovorus GRL1118 (-) 1686731
L. johnsonii BS15 (-) 520655
L. acidophilus NCFM (-) 1754392
L. delbrueckii spp delbrueckii KCTC13731 (-) 1710225
L. paracollinoides TMW1.1994 725916
L. helveticus DPC4571 (-) 1762888
L. helveticus H10 (-) 1836550
L. delbrueckii spp bulgaricus ATCC11842_b (-) 1668565
L. johnsonii NCC533 936499
L. koreensis 26-25
2279515
L. ginsenosidimutans EMML3041 (-) 1447493
L. delbrueckii spp sunkii JCM17838 (-) 52776
L. delbrueckii spp bulgaricus 2038_a (-) 1652844
L. delbrueckii spp bulgaricus ND04_b 1173130
L. delbrueckii spp bulgaricus ATCC11842_a (-) 1626309
L. delbrueckii ATCC BAA 365_a (-) 1641495
Group 3
L. delbrueckii spp indicus JCM15610 (-) 1693498
L. paracasei KL1 413417
L. curvatus WiKim38 477397
L. sakei spp sakei 23K (-) 1475623
L. salivarius UCC118_a (-) 992876
L. plantarum ZS2058_a 296902
L. planatarum TS12_a 307245
L. plantarum HFC8_a (-) 570533
L. plantarum NCU116_c 2560950
L. plantarum WCFS1_d (-) 1892017
L. planatarum P-8 (-) 1752180
L. plantarum DOMLa_b (-) 1862188
L. plantarum JDM1_b (-) 1861125
L. plantarum ZS2058_b (-) 1072895
L. plantarum LZ206_a 639574
L. planatarum Zhang-LL (-) 1713334
L. plantarum ST-III_c (-) 1858725
L. plantarum RI-113_a 859293
L. plantarum MF1298_b (-) 1814407
L. plantarum LY-78 142612
L. plantarum KP_a 2176961
L. planatarum JBE245_c (-) 1913909
L. plantarum DF_a 2552100
L. plantarum CLP0611_b (-) 1827459
L. plantarum CAUH2_c (-) 1861691
L. plantarum C410L1 709677
L. planatarum 16_b (-) 1760288
L. plantarum B21_b (-) 1917216
L. plantarum ZJ316_c (-) 2135771
L. plantarum WCFS1_b 1100812
L. buchneri NRRL B-30929 (-) 37099
L. fermentum NCC2970 1037670
L. helveticus H9 (-) 1610794
L. reuteri TD1 (-) 1212387
L. curvatus FBA2 (-) 1491168
L. kunkeei MP2 857171
L. fermentum SNUV175_a (-) 887835
Group 4
L. plantarum MF1298_a 1025968
L. plantarum ZJ316_b 1311520
L. plantarum WCFS1_c 1123133
L. plantarum ST-III_b 1082952
L. plantarum KP_b (-) 2953603
L. plantarum NCU116_a (-) 135918
L. planatarum TS12_b (-) 1088231
L. plantarum LZ206_b (-) 1421303
L. planatarum JBE245_b 1131088
L. plantarum CAUH2_b 1123061
L. plantarum DF_b (-) 3328741
L. plantarum CLP0611_a
1048940
L. plantarum B21_a
1120443
L. plantarum WCFS1_a 1088256
L. salivarius CICC23174 717453
L. salivarius UCC118_b (-) 1626392
Group 5
L. planatarum JBE245_a 1084625
L. planatarum TS12_c (-) 1110320
L. plantarum LZ206_c (-) 1452735
L. plantarum KP_c (-) 2984338
L. plantarum CAUH2_a 1092467
L. plantarum ST-III_a 1052654
L. plantarum ZJ316_a 1282872
L. plantarum DF_c (-) 3359476
L. plantarum RI-113_b (-) 1607531
Group 6
L. casei BDII (-) 2141734
L. casei BL23 (-) 2149759
L. casei W56 (-) 2147995
L. casei LC2W (-) 2110848
L. casei LOCK919 (-) 2173729
L. rhamnosus GG (-) 2088780
L. casei spp casei ATCC393_b (-) 1922306
L. rhamnosus Lc705_b (-) 2061488
L. rhamnosus LOCK900_b (-) 1962778
L. casei Zhang_b (-) 1949787
L. curvatus WiKim52 944888
L. fermentum CECT5716_a 124098
L. fermentum IFO3956_a 123701
L. brevis KB290 (-) 1597541
L. amylophilus DSM20533 1367961
L. reuteri I49 (-) 740327
L. planatarum 16_a 20336
L. plantarum DOMLa_a 1106649
L. plantarum JDM1_a 1106708
L. plantarum NCU116_b (-) 156626
Group 7
L. plantarum HFC8_b 76919
L. casei spp casei ATCC393_a (-) 1865986
L. rhamnosus LOCK900_a (-) 1908032
L. rhamnosus Lc705_a (-) 2006878
L. casei Zhang_a (-) 1890597
L. paracasei L9 (-) 2089030
L. rhamnosus LOCK908 (-) 2024356
L. paracasei N1115 771874
Group 8
L. reuteri IRT (-) 1414598
L. paracasei spp paracasei JCM8130 (-) 2020130
L. reuteri JCM1112 (-) 1459664
L. reuteri I5007_a 629655
L. reuteri ZLR003_a 1857205
L. oris J-1_b 2389557
L. fermentum CECT5716_b (-) 1585070
L. fermentum IFO3956_b (-) 1583925
L. fermentum SNUV175_b 1555781
L. fermentum F6_b (-) 1566625
L. lindneri TMW1.481 582159
L. casei ATCC334 241433
epsA
Phosphoregulatory module
epsE
GT
epsX
epsY
Precursor Biosynthesis
Decoration
Other EPS related function
Other transcriptional regulators
Other cell surface related
Transposase
Unknown function
Unrelated function
Scale
1 kb
